# Penile necrosis by calciphylaxis leading to gangrene in a patient with chronic renal failure on dialysis: A case report

**DOI:** 10.1016/j.ijscr.2020.04.091

**Published:** 2020-05-11

**Authors:** Youssef Kouiss, Mohammed Aynaou, Amine El Houmaidi, Tarik Mhanna, Yacoub Ahmed, Abdelghani Ouraghi, Achraf Miri, Amal Bennani, Ali Barki

**Affiliations:** a Department of Urology, Mohamed VI University Hospital, Oujda, Morocco; b Department of Pathology, Mohamed VI University Hospital, Oujda, Morocco

**Keywords:** CUA, Calcific uremic arteriolopathy, CKD, Chronic kidney disease, CRF, Chronic renal failure, CT, Computerized tomography, HB, Haemoglobin, MRI, Magnetic resonance imaging, PTH, Parathormone, Calciphylaxis, Calcific uremic arteriolopathy, Fournier’s gangrene, Penis necrosis, Penectomy, Case report

## Abstract

**Introduction:**

Penile calciphylaxis is a rare disease whose diagnosis is not easy. It is associated with chronic renal failure. It is a result of media calcification and blood vessels’ fibrosis such as penile arteries that eventually lead to gangrene formation in extremities and penis. Calcific uremic arteriolopathy is commonly associated with secondary hyperparathyroidism and high level of calcium and phosphate.

**Presentation of case:**

In this paper, we report a case of penile necrosis induced by calciphylaxis associated with chronic renal failure and diabetic macroangiopathy. We performed a partial and total amputation in two stages because of the wound infection. The patient died one week after a total penectomy.

**Discussion:**

The skin biopsy is the gold standard for the diagnosis despite being a risky process. In most cases, appropriate medical management is advocated. The treatment of this pathology is still controversial.

**Conclusion:**

The management of this rare situation is controversial, and its diagnosis is still difficult due to the scarcity of reported cases.

## Highlights

• Penile necrosis by calciphylaxis is a rare disease.
• The diagnosis of CUA is often difficult.
• The Management of this rare condition is still a matter of debate.

## 1 Introduction

Penile necrosis is a very rare condition in that most of its cases are associated with systemic calciphylaxis. In this situation, the penis rarely becomes ischemic because it receives blood through 3 arterial pathways. Progressive cutaneous necrosis may be caused by arteriosclerosis that is accompanied by calcification of blood vessels [1]. Calcific uremic arteriolopathy (CUA) or calciphylaxis is uncommon and a deadly disease in patients with chronic renal failure. Several risk factors are identified including sex (women seem to be more affected than men), Caucasian ethnicity, duration of chronic kidney disease, seniority on dialysis, diabetes, hepatopathies, obesity and autoimmune diseases are other recognized risk factors for this rare entity [2]. In addition, disorders in phosphocalcic metabolism are also involved, and this includes secondary hyperparathyroidism, increased phosphocalcic product, hyperphosphatemia and vitamin D deficiency. A few cases of penile necrosis have been reported so far. The incidence of penis calciphylaxis is reported to be 6%, mainly in diabetic patients with chronic renal failure and abnormal levels of calcium and phosphorus [3,4]. The diagnosis of CUA is often difficult and is based on skin biopsy [5]. Until the present time, there are no well-established management protocols for this disease. This work has been reported in line with the SCARE criteria [6].

## 2 Case report

**Fig. 1:**
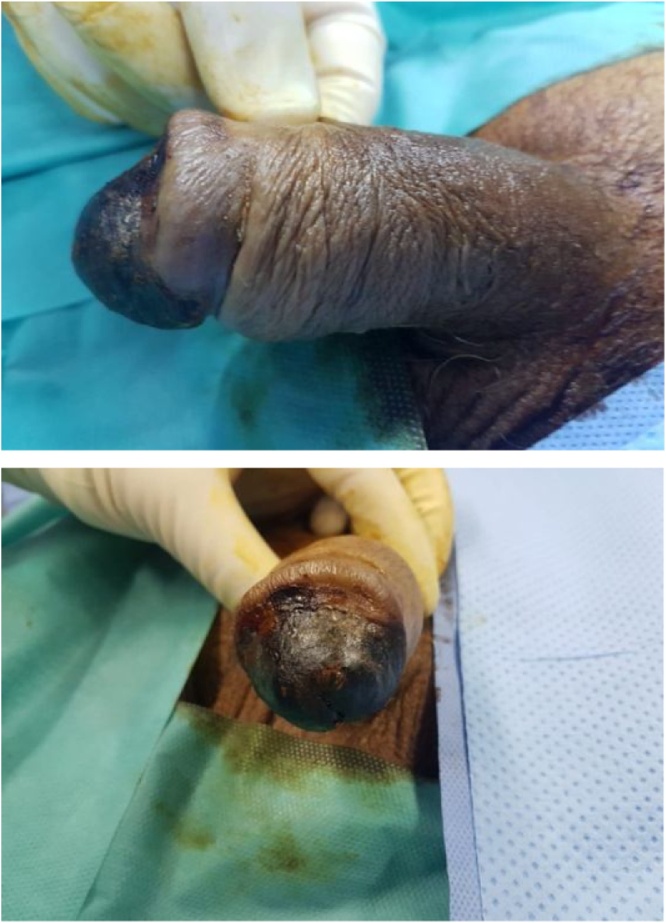
Preoperative appearance of penile necrosis.

We report a case of an 80-year-old man with a history of arterial hypertension and type 2 diabetes who uses an insulin therapy since 2012. The patient used to suffer from terminal renal insufficiency (stage 5) for 10 years treated with hemodialysis (3 sessions per week). Also, the patient had a long history with smoking (at a rate of 2 packs/day for 30 years) suspended 1 year ago. Eight months ago, the patient was admitted for pain and changes in color in the left lower limb. Evaluated by the vascular department, it was detected that there are many necrosis in his bilateral lower limbs. The patient was treated by surgical amputation of his left lower limb in March 2019, and nine months later, the patient’s right lower limb was amputated. Two months later, the patient was referred to our department because of a severe penile pain and necrosis. On clinical examination, there were necrotic ulcer and blackish discoloration of the glans (Fig. 1). The patient had a urinary tract infection, the urine culture found E coli, so he started antibiotic treatment for 10 days by quinolones (ciprofloxacin) 500 mg twice a day).

**Fig. 2:**
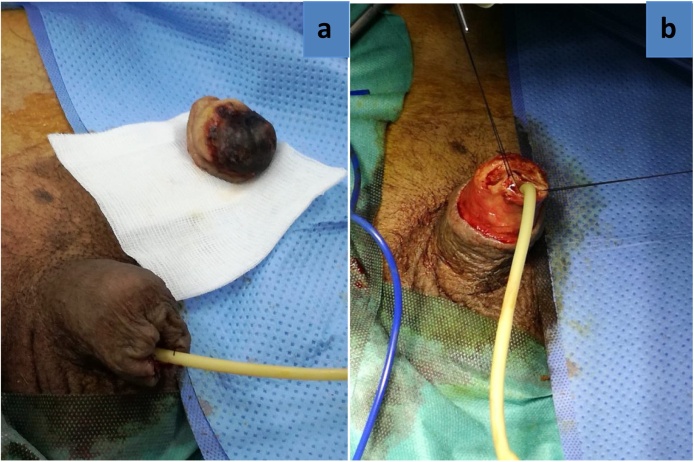
(a) Immediate post-op of the partial penectomy and (b) a trans-surgery of partial penectomy.

**Fig. 3:**
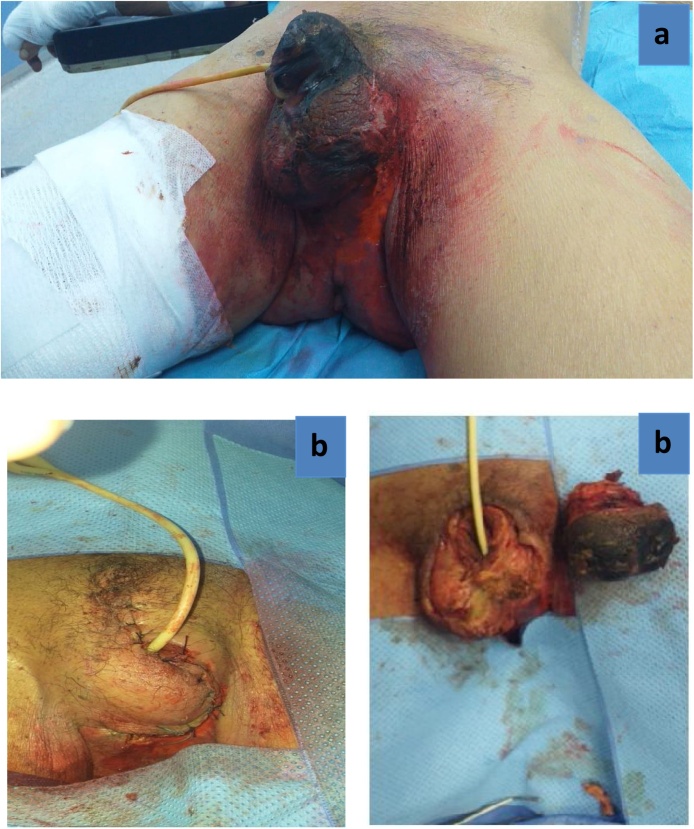
(a) Wound infection with wet gangrene and (b) post-operatory total penectomy.

**Fig. 4:**
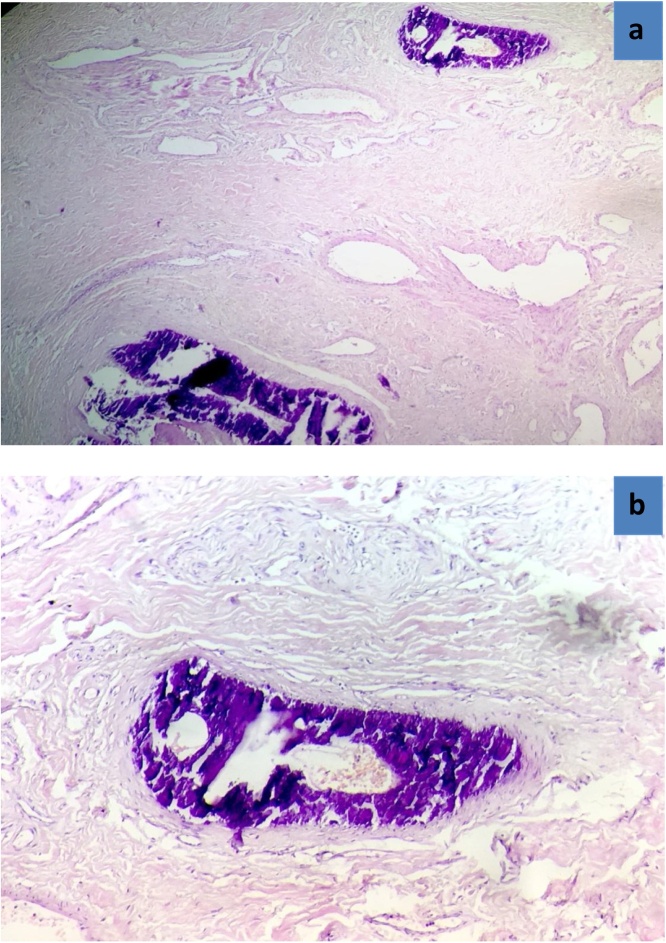
(a) Microphotography showing important calcium deposits in vessels lumen with intimal fibroblastic proliferation (HE; 100X) and (b) microphotography at higher magnification showing the marked calcium deposits with an advanced diabetic microangiopathy (HE; 200X).

Due to a severe penile pain, the patient was programmed for partial penectomy (Fig. 2a) after taking his permission as well as that of his family. In the trans-surgery, the absence of vascular flow was observed in the penile arteries and in the deep dorsal vein (Fig. 2b).The following management was based on a daily wound care, debridement for the next 2 weeks and an antibiotic-based treatment for a second episode of urinary tract infection. So, he received imipenem 500 mg two times per 10 day. In the first postoperative days, the patient’s state shows recovery, but from the 14th day of this intervention, the surgical wound remained open with little serohematic secretion, as well as infected tissues shows poor progression that led to Fournier’s gangrene (Fig. 3a). Then, a total penectomy was performed through removing a part of his left scrotum (Fig. 3b). An arteriography was performed during his hospital stay in the vascular department which showed multiple vascular calcifications notably at the level of the internal iliac artery and its branches. During laboratory assessment, the creatinine and urea levels in the blood were higher than the laboratory values: Creatinine 78 mg/L, urea 82.2 g/L. The other results were like the following: Serum Ca was slightly decreased to 83 mg/Land serum phosphate was high reaching 55 mg/L. In addition to that, parathyroid hormone level was not recorded, and vitamin D was deficient lowering to10.8 ng/mL. Elevated inflammatory markers were noticed including C-reactive protein and leukocytes because of his urinary tract infection. Haemoglobin (Hb) was only 7.2 g/dL due to renal anemia by end-stage renal failure. The histopathological analysis found an advanced diabetic microangiopathy accompanied by significant calcium deposits in vessels lumen with intimal fibroblastic proliferation (Fig. 4a and b).

## 3 Discussion

CUA manifests by purplish plaques with a necrotic center, delimited by an extremely painful pink rim, and hardened subcutaneous nodules [5]. It is observed in 1–4% of patients with CRF. Lesions typically occur in the fatty areas such as abdominal fat, flanks, buttocks and inner thighs [5]. In rare cases, CUA affects internal organs (digestive tract, lungs, heart or eyes) and distal ends [5] (penis in our case). The pathophysiology of CUA is complex and is not yet fully understood. Two stages are necessary for the development of pathology: 1) a long and insidious process leading to calcifications of the arteriole media and intimal fibrosis and 2) an acute process of thrombotic occlusion resulting from progressive calcification and endothelial dysfunction [5]. The diagnosis of CUA is often difficult. Indeed, there is no biochemical analysis or radiological examination allowing a precise diagnosis. Various imaging modalities have been studied, but none of them allows us to make the diagnosis of CUA out of certainty. The gold standard to confirm this diagnosis is the skin biopsy [5]. Indeed, it is likely to cause additional trauma, create a new focus of necrosis and leads to ulceration that aggravate the skin lesions which are already present. For this reason, the biopsy is reserved for patients whose diagnosis is uncertain [7]. Histopathological analysis of CUA wound shows calcifications of the tunica intima and media of the penis, calcifications of perivascular soft tissue, as well as necrosis of the epidermis.

In previously reported cases [[8], [9], [10], [11], [12], [13], [14], [15]] (Table 1), all patients including the present case had chronic kidney disease (78% were reported on dialysis) and diabetes mellitus which could be a predisposing factor [16]. Seven reported cases, including our patient’s case, had a high blood pressure and two of them had a long history with smoking [[8], [9], [10], [11], [12], [13], [14], [15]]. In our case, other risk factors were found such as obesity and anticoagulant treatment that may worsen pre-existing atherosclerosis. In our case, the histopathological diagnosis of calciphylaxis shows diabetic microangiopathy that is accompanied by vascular calcification of both the tunica intima and media of blood vessels with intimal fibroblastic proliferation. In other cases [[8], [9], [10], [11], [12], [13], [14], [15]], the histopathological diagnosis was performed in 6 patients including our case and 8 patients who received at least one radiological assessment. In our case, the patient received an arteriography that showed multiple vascular calcifications at the level of the internal iliac artery and its branches. The rest of the imaging exams were not performed. In a review about the use of radiological investigations in the management conducted by Campbell et al., it was shown that the used radiological exams, including Doppler ultrasound, MRI and CT, may be helpful in choosing early surgical intervention due to the rapid extent of necrosis [10].

**Table 1:** Most reported cases of penile necrosis by calciphylaxis in a diabetic patient with chronic renal disease in literature in the last 10 years.

References	Year	Age	Diabetes	Chronic renal disease	High blood pressure	Smokingstatus	Diagnostic tools	Medical therapy	Surgical treatment	Outcomes
I [8]	2014	61	Type 1 diabetes	Hemodialysis since 2012	Yes	Yes	Histopathalogy	Antibiotics	Total penectomy with perineal urethrostomy	Died
II [9]	2015	76	Diabetes	Hemodialysis for 2 years now	No	No	CT	–	Endovascular therapy	Alive
III [10]	2017	46	Diabetes	Hemodialysis for 5 years now	Yes	No	Doppler ultrasound + CT scan + histopathology	–	Total penectomy with penoscrotal urethrostomy	Alive
IV [11]	2017	64	Diabetes	End stage renal disease	Yes	No	CT	–	Partial penectomy	Alive
V [12]	2018	47	Type 2 diabetes	On hemodialysis	Yes	No	MRI of the pelvis + CT	Palliative care	Conservative treatment + Suprapubic catheter	Not reported
VI [13]	2018	60	Type 1 diabetes	Chronic renal disease stage V	No	No	CT urography + histopathology	Systemic antibiotics + Sodium thiosulfate	Partial penectomy	Alive
VII [14]	2019	65	Type 2 diabetes	Hemodialysis	Yes	Yes	–	–	Conservative treatment	Alive
VIII [15]	2019	53	Type 1 diabetes	Hemodialysis for 2 years now	Yes	No	Doppler ultrasound + histopathology	Hyperbaricoxygen + systemic antibiotic	Partial penectomy	Alive

The treatment of this disease is still controversial. Some authors mention that there is conservative management with antibiotic therapy and local debridement. However, other authors suggest performing partial or total penectomy. In our case, we were not able to have a conservative management due to the rapid development of penile necrosis and a severe pain, a partial penectomy was selected for the present patient with antibiotics. The patient was responding positively to the treatment in the first days. However due to the worsening of his situation that, 2 weeks later, led to Fournier’s gangrene, a total penectomy was carried out. The survival was higher in patients who had undergone partial penectomy (37.5%) than in those treated with total penectomy (12.5%). Endovascular revascularization [9] and conservative treatment [14] appeared to play an important role in the penile’s wound healing.

Based on the data of our current case and the recent literature reviews, the management of this disease can be personalized based on the availability of the procedures of diagnosis and the experience of each team.

## 4 Conclusion

Penile necrosis is a rare disease that is associated with chronic kidney disease. It is related with the presence of systemic calciphylaxis and diabetes as a cofactor in its pathophysiology. We reported a case of penile necrosis caused by calciphylaxis associated with CKD and diabetic macroangiopathy with long history with smoking and arterial hypertension. The patient underwent surgical regularization of the necrosis in two stages because of his accelerated progression of the necrosis that led to Fournier's gangrene a few days after the partial penectomy.

## Declaration of Competing Interest

No.

## Funding

No.

## Ethical approval

The ethical approval has been exempted by our institution.

Not required for this case report.

The patient gave written permission to publish his case findings before he passed away.

## Consent

The patient gave written permission to publish his case before he passed away.

## Author contribution

Youssef Kouiss, Mohammed Aynaou, Tarek Mhanna, Amine El houmaidi wrote the article.

Yacoub Ahmed, abdelghani Ouraghi participated in the patient’s care.

Achraf Amiri, Amal Bennani were involved in the histopathological analysis.

Ali Barki supervised the article writing.

## Registration of research studies

Not required.

## Guarantor

Youssef Kouiss.

Ali Barki.

## Provenance and peer review

Not commissioned, externally peer-reviewed.
